# Perfusion Enhancement via Continuous Brachial Plexus Block to Prevent Vascular Insufficiency in Replanted Digits: A Prospective, Randomized Controlled Trial

**DOI:** 10.3390/jcm14186591

**Published:** 2025-09-18

**Authors:** Yang Xu, Fang Xie, Yan Wang, Jie Chen, Shenghe Liu, Tao Xu

**Affiliations:** 1 Department of Anesthesiology, Shanghai Sixth People’s Hospital Affiliated to Shanghai Jiao Tong University School of Medicine, 600 Yishan Road, Shanghai 200233, China; zipyang@126.com (Y.X.); fyyxz2015@163.com (F.X.); wangyanxq1989@163.com (Y.W.); 2 Department of Ultrasound Medicine, Shanghai Sixth People’s Hospital Affiliated to Shanghai Jiao Tong University School of Medicine, 600 Yishan Road, Shanghai 200233, China; 3 Department of Orthopedics, Shanghai Sixth People’s Hospital Affiliated to Shanghai Jiao Tong University School of Medicine, 600 Yishan Road, Shanghai 200233, China

**Keywords:** digit replantation, continuous brachial plexus block, vascular insufficiency, perfusion, skin temperature

## Abstract

**Background/Objectives:** Pain-induced vasoconstriction and thrombosis cause vascular insufficiency, a major etiology of early necrosis in replanted digits. While systemic intravenous analgesia (SIVA) carries significant side effects, continuous brachial plexus block (CBPB) provides analgesia with vasodilation. Amidst uncertainties regarding distal vascular recanalization and sympathetic denervation, whether CBPB’s hemodynamic effects translate into improved perfusion and outcomes in replanted digits remains unknown. This randomized controlled trial compared the effects of CBPB versus SIVA on digit perfusion, vascular insufficiency, and survival rates post-replantation. **Methods:** After screening 113 patients, 55 patients (71 digits) were ultimately randomized and analyzed: the CBPB group (*n* = 27, 36 digits) received 0.2% ropivacaine infusion at 5 mL/h; the SIVA group (*n* = 28, 35 digits) received intravenous parecoxib 20 mg twice daily with supplemental tramadol for visual analog scale (VAS) scores > 3. The primary outcome was digital skin temperature trajectory measured at 0, 12, 24, 36, and 48 h postoperatively. Secondary outcomes included Doppler-quantified combined volumetric flow rate of the radial and ulnar arteries (RA-UA VFR) at identical timepoints, VAS scores, vascular insufficiency incidence, and 7-day digit survival. **Results:** CBPB significantly enhanced perfusion in replanted digits at all postoperative timepoints, with digital skin temperature peaking at 48 h (32 ± 1.6 °C vs. 31 ± 1.1 °C; *p* < 0.001) and RA-UA VFR peaking at 24 h (4.0 ± 0.83 vs. 1.8 ± 0.51 mL/s; *p* < 0.001) versus SIVA. Concurrently, CBPB provided superior analgesia (VAS 0.52 ± 0.51 vs. 1.9 ± 1.0; *p* < 0.001) and significantly reduced 48-h vascular insufficiency incidence (8.3% vs. 29%; *p* = 0.028). No significant difference was observed in seven-day survival rates between groups (97% vs. 91%; *p* = 0.329). **Conclusions:** CBPB significantly enhanced perfusion in replanted digits and reduced the incidence of vascular insufficiency, despite not conferring additional survival benefits.

## 1. Introduction

Digit amputations resulting from occupational injuries in the furniture, electronics assembly, and construction sectors can be effectively addressed by replantation surgery, which restores osteotendinous integrity and neurovascular continuity to optimize functional recovery and cosmetic outcomes [1]. The success of digital replantation depends not only on the intraoperative microsurgical anastomosis but also critically on maintaining the patency of the anastomosed vessels postoperatively [2]. During the critical 48-h postoperative period, local pain triggers sympathetically mediated vasospasm and subsequent thrombosis, disrupting the delicate equilibrium of digital microcirculation. This initiates a pathological cascade culminating in vascular insufficiency, defined as impaired perfusion characterized by critically diminished arterial inflow or venous outflow dysfunction [3]. This imbalance necessitates urgent salvage procedures to mitigate impending tissue necrosis [4]. The resulting pathophysiology underscores the critical need for dual application of postoperative analgesia and vasodilatory protocols to optimize replantation survival.

In the absence of clinical guidelines for postoperative management of replantation, systemic intravenous analgesia (SIVA) employing nonsteroidal anti-inflammatory drugs (NSAIDs) carries inherent risks of complications such as gastrointestinal ulceration and renal impairments, while opioids inherently cause respiratory depression, nausea and vomiting [5]. In contrast, continuous brachial plexus block (CBPB) is being increasingly adopted as the preferred postoperative pain management strategy by a growing number of replantation centers due to its sympathectomy-induced vasodilatory effects [6,7,8]. Clinical observations by Clifford et al. [9] and Stevens et al. [10] document enhanced extremity perfusion following upper limb replantation with CBPB. Nevertheless, the increased blood flow of proximal anastomotic artery does not necessarily enhance perfusion in replanted digits, given the uncertainties surrounding vascular recanalization and sympathetic denervation in distal anastomotic vessels. Moreover, whether augmented perfusion reduces vascular insufficiency incidence and further improves survival rates remains uncertain given insufficient evidence-based research.

In this randomized controlled trial comparing CBPB with SIVA after digital replantation, we assessed the quantitative improvement in blood flow to the affected hand with CBPB, determined whether this hemodynamic enhancement translated to improved perfusion of replanted digits, and examined the effects of CBPB on vascular insufficiency incidence and survival rates of replanted digits.

## 2. Patients and Methods

### 2.1. Study Design and Setting

This open-label prospective randomized controlled trial was conducted at Shanghai Sixth People’s Hospital, which is the largest replantation center in Shanghai. It was prospectively registered in the Chinese Clinical Trial Registry on 12 June 2019 (Trial NO. ChiCTR1900023796). Written informed consent was obtained from all patients. Inclusion criteria comprised adults aged 18–65 years with clinically and radiographically (tissue separation, ischemia; X-ray) confirmed acute digital amputations, assigned an American Society of Anesthesiologists (ASA) physical status classification of I–II, and scheduled for microsurgical revascularization. Exclusion criteria included chronic vascular comorbidities (peripheral artery disease, coagulopathies, diabetes), pregnancy, cognitive impairment, and preexisting ipsilateral upper extremity neuropathy.

### 2.2. Randomization

Patients admitted to the emergency department were randomized 1:1 to the CBPB group or SIVA group based on postoperative pain management strategies, using sequentially numbered, opaque, sealed envelopes containing computer-generated randomization assignments.

### 2.3. Anesthetic Protocol and Interventions

All patients received standardized anesthesia comprising ultrasound-guided costoclavicular brachial plexus block (BPB), with dexmedetomidine sedation (intravenous infusion dose at 0.2–0.7 μg/kg/h).

**Figure 1:**
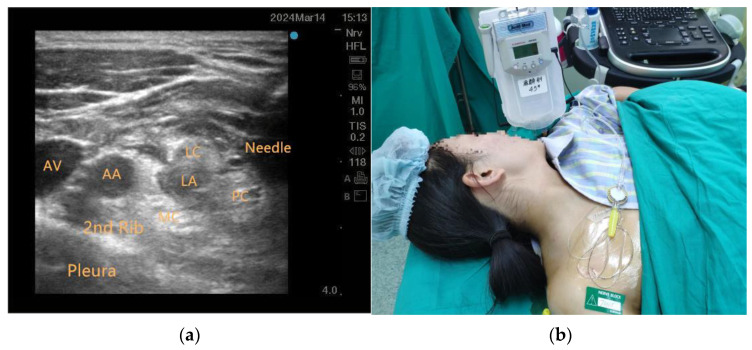
Ultrasound-guided BPB and postoperative analgesia in CBPB group. (**a**) Ultrasound image of the costoclavicular BPB; (**b**) PCA-connected catheter secured to thoracic skin; AA = axillary artery; AV = axillary vein; LC = lateral cord; MC = medial cord; PC = posterior cord; LA = local anesthetic.

Following baseline monitoring, a dexmedetomidine loading dose (1 μg/kg over 10 min) was administered prior to BPB performed using an ultrasound system (S-NERVE, FUJIFILM SonoSite Inc., Bothell, WA, USA). A high-frequency linear probe (6–15 MHz) was placed transversely at the clavicular midpoint and adjusted caudally to visualize brachial plexus cords lateral to the axillary artery. The needle was advanced through the lateral/posterior cords to inject 30 mL 0.5% ropivacaine, circumferentially enveloping all cords under real-time guidance (Figure 1a). Patients allocated to the CBPB group underwent catheter insertion (Contiplex, B. Braun Melsungen AG, Melsungen, Germany) following BPB. This catheter was connected to a patient-controlled analgesia pump (AutoMed 3300, Ace Medical Co., Ltd., Seoul, Republic of Korea), programmed to administer 0.2% ropivacaine (Naropin, AstraZeneca AB, Södertälje, Sweden) via a continuous basal infusion of 5 mL/h, with patient-activated boluses of 5 mL available at 15-min lockout intervals for 48 h postoperatively (Figure 1b). Conversely, patients in the SIVA group received scheduled intravenous parecoxib (Dynastat, Pfizer Inc., New York, NY, USA) 20 mg twice daily, supplemented by intravenous tramadol (Tramal, Grünenthal GmbH, Aachen, Germany) 100 mg on demand for visual analogue scale (VAS) scores > 3, adhering to a minimum 6-h dosing interval.

### 2.4. Postoperative Management

All patients were transferred to our specialized replantation intensive care unit staffed with microsurgical nurses for standardized postoperative care. Room temperature was maintained at 22–25 °C, and a strict 72-h bed rest regimen with limb elevation was implemented, supplemented by bladder catheter removal on postoperative day 1, early transition to oral intake, and mandatory smoking abstinence for ≥1 month. Antibiotic prophylaxis involved a single preoperative dose discontinued within 24 h [11]. Low molecular weight heparin (LMWH) (Clexane, Sanofi, France) was initiated postoperatively for thromboprophylaxis, with doses titrated to achieve an activated partial thromboplastin time (APTT) of 1.5 to 2.5 times the normal range [4]. Replanted digits were dressed with non-constrictive bandages exposing fingertips for perfusion monitoring. Hourly evaluations, conducted by two trained microsurgical nurses with ≥5 years of replantation care experience (with physician consultation for equivocal cases), assessed skin color, turgor, capillary refill, and temperature. Salvage revascularization was indicated if arterial insufficiency failed to resolve within 1 h of conservative measures (warming, analgesic optimization), venous congestion worsened despite medical therapy (leeching, heparinization) over 2 h, or ongoing ischemia threatened digit viability (persistent pallor/cyanosis with no Doppler signal).

### 2.5. Primary and Secondary Study Outcomes

The primary outcome was the trajectory of digital skin temperature—a key clinical indicator of capillary perfusion—assessed at 0 (post-closure), 12, 24, 36, and 48 h postoperatively. Secondary outcomes comprised: (1) the combined volumetric flow rate of the radial and ulnar arteries (RA-UA VFR), quantified by Doppler ultrasonography at identical timepoints as a surrogate measure of blood flow in the affected hand; (2) VAS scores assessing analgesic efficacy; (3) the incidence of vascular insufficiency, defined as arterial insufficiency (concomitant presence of pallor, ≥3 °C temperature differential versus the contralateral digit, and delayed capillary refill >2 s) or venous congestion (simultaneous presence of purplish discoloration and rapid capillary refill <1 s) [2]; and (4) the 7-day survival rate of replanted digits, defined by viable tissue exhibiting capillary refill <2 s and a ≤1 °C temperature differential compared to the contralateral digit.

### 2.6. Outcome Measures

**Figure 2:**
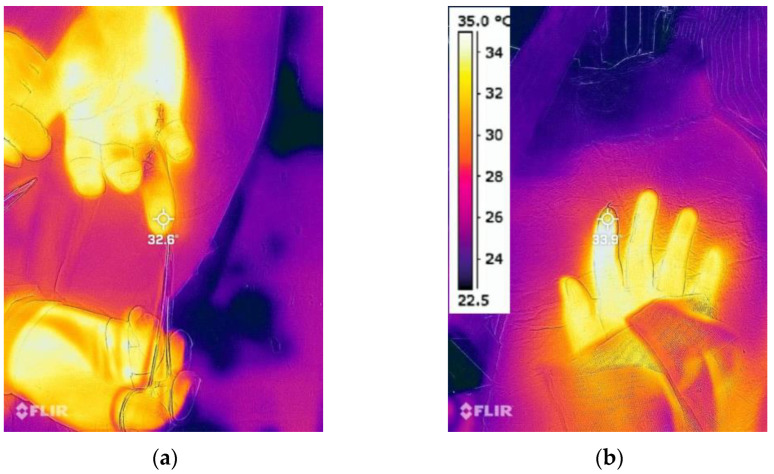
Infrared thermographic images of the replanted digits. (**a**) Skin temperature immediately after surgical closure; (**b**) Skin temperature 48-h after surgery.

Digital skin temperature was assessed via an infrared thermographic camera (FLIR one pro, FLIR Systems Inc., Wilsonville, OR, USA) fixed 1.3 m vertically above the digits, providing thermal sensitivity ≤ 0.1 °C and ±3% accuracy. During the 1-min recordings (spatial resolution: 160 × 120 pixels), the affected hand was immobilized (Figure 2). Each day, prior to any patient measurement, the camera was validated against a NIST-traceable black-body source (Hart Scientific 7012, 35 °C); if any pixel deviated >0.2 °C from the reference value, the factory-calibration file was re-uploaded via FLIR Tools software (version 4.1.0, FLIR Systems Inc., Wilsonville, OR, USA).

**Figure 3:**
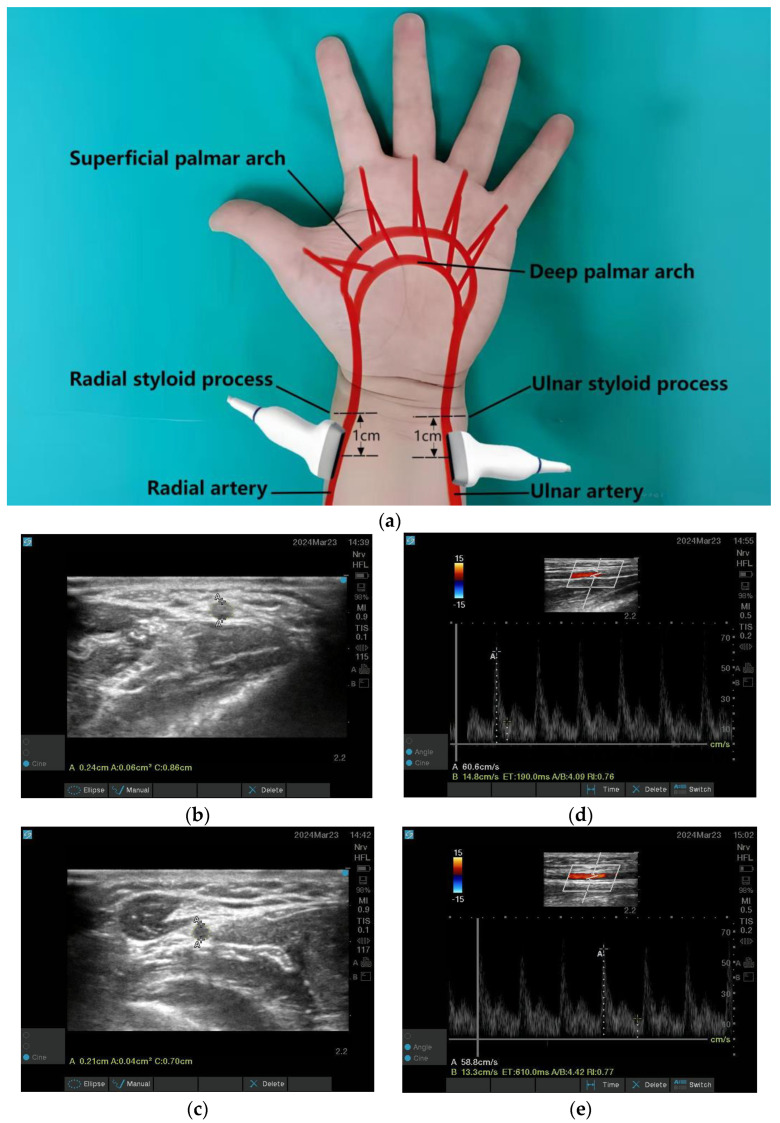
Doppler Ultrasonography assessment of radial-ulnar arterial hemodynamics. (**a**) Palmar arterial schematic and Ultrasonographic probe positioning; (**b**,**c**) Ultrasound image used to measure the cross-sectional area of the radial artery and ulnar artery; (**d**,**e**) Longitudinal duplex Doppler spectral waveform used to measure time-averaged mean velocity and resistance index for the radial artery and ulnar artery.

Anatomically, the proper digital arteries, originating from the radial artery (RA) and ulnar artery (UA) via palmar arch anastomoses, form the direct arterial supply to digits [12] (Figure 3a). Therefore, this study adopted the RA-UA VFR as a surrogate measure of blood flow in the affected hand, reflecting the perfusion capacity of the proximal anastomotic artery. Measurements of arterial cross-sectional area (CSA), time-averaged mean velocity (TAMV), and resistance index (RI) were performed 1 cm proximal to the radial/ulnar styloid process (Figure 3a) using a color Doppler ultrasound system (S-NERVE, FUJIFILM SonoSite Inc., Bothell, WA, USA), with hemodynamics quantified through its integrated software. Short-axis CSA images were obtained with the probe perpendicular to the arterial axis (Figure 3b,c), while long-axis TAMV measurements required parallel alignment (Figure 3d,e). VFR was calculated as Q = A × V (CSA [cm^2^] × TAMV [cm/s]), with RA-UA VFR defined as the sum of RA and UA values. Daily calibration followed the manufacturer’s protocol: the built-in quality-assurance phantom (CIRS 040GSE) was scanned; if colour-flow or pulsed-wave velocity deviated >5% from the reference value the system auto-corrected and stored a new baseline. All CSA and velocity measurements were subsequently obtained with the same gain/scale settings validated in the phantom.

VAS assessments were conducted every 3 h during the initial 48 postoperative hours, with patients self-reporting pain intensity by marking a position on a 10-cm horizontal scale ranging from “no pain” (0) to “worst imaginable pain” (10).

### 2.7. Sample Size Calculation

The sample size was determined based on the primary outcome of intergroup differences in postoperative digital skin temperature between the CBPB and SIVA groups. A pilot study (*n* = 20) demonstrated a mean temperature difference of 1.7 °C (CBPB: 32 ± 1.2 °C versus SIVA: 30 ± 1.1 °C; mean difference 1.7, 95% CI 0.92–2.5; *p* = 0.012) within 48 h postoperatively. To detect this clinically significant ΔT (≥1.20 °C) with a two-tailed independent t-test (α = 0.05, β = 0.20, power = 80%), GPower 3.1 (Heinrich Heine University, Germany) calculated a minimum requirement of 25 participants per group, assuming a pooled standard deviation (SD) of 1.20 °C. Accounting for 10% attrition, 27 CBPB and 28 SIVA participants were enrolled. Post hoc power analysis confirmed 89% power to detect the observed intergroup difference, validating the adequacy of the final sample size for hypothesis testing.

### 2.8. Ethical Approval

The study protocol received ethical approval from the Ethics Committee of Shanghai Sixth People’s Hospital, Shanghai, China (Approval No. 2019-060-1, approval date: 18 September 2019).

### 2.9. Statistical Analysis

Statistical analyses were performed using SPSS 28.0 (IBM, USA) and GraphPad Prism v9 (GraphPad Software LLC, USA) with a significance threshold of α = 0.05. Continuous variables (VAS, skin temperature, RA-UA hemodynamics) were tested for normality (Shapiro–Wilk, W > 0.90). Normally distributed data (mean ± SD) underwent repeated-measures ANOVA with Greenhouse-Geisser correction and Bonferroni post hoc tests. Significant Group × Time interactions (*p* < 0.05) prompted Bonferroni-adjusted t-tests at individual timepoints. To account for repeated measurements of skin temperature within the same patient, generalized estimating equations (GEE) with an exchangeable correlation structure were additionally employed with group and time as fixed effects. Missing data from secondary surgeries were imputed via last observation carried forward (LOCF). χ^2^/Fisher’s exact tests for categorical outcomes were performed at the digit level to verify robustness. To address potential non-independence of multiple digits within a single patient, GEE with an exchangeable correlation structure were employed for binary outcomes (vascular insufficiency incidence, secondary revascularization and survival rates).

## 3. Results

### 3.1. Participants’ Baseline Data

**Figure 4:**
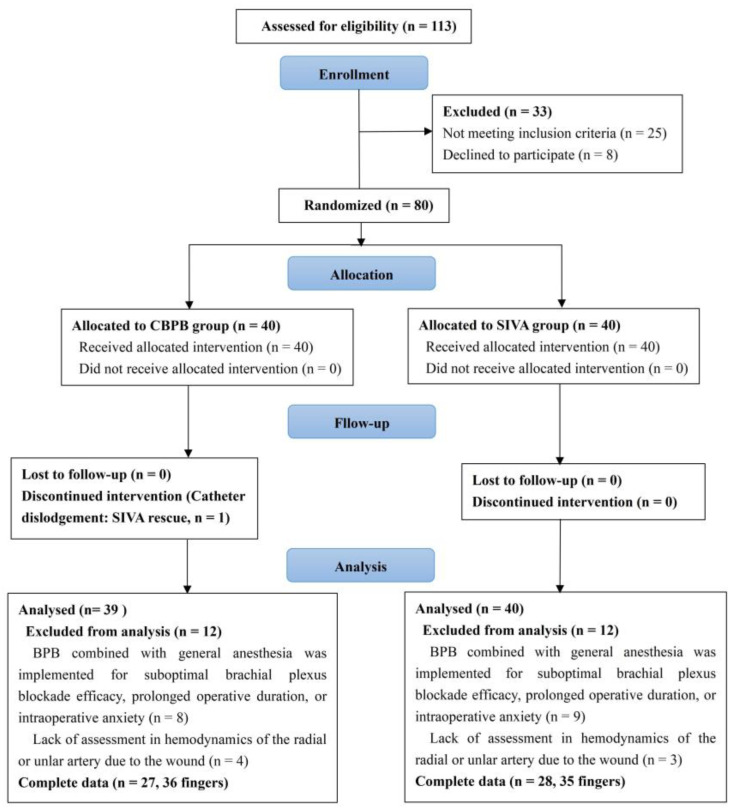
CONSORT diagram of participant selection.

From December 2021 to December 2024, 113 finger amputation patients received treatment. After excluding 33 patients due to failure to meet criteria and voluntary withdrawal, the remaining 80 patients were randomly assigned to the CBPB group and SIVA group, with 40 patients in each group. 1 patient in the CBPB group was excluded due to catheter dislodgment, and both groups had 12 patients each excluded for reasons including anesthesia protocol deviations and incomplete hemodynamic assessments. Ultimately, 27 patients (36 digits) in CBPB and 28 patients (35 digits) in SIVA with complete data were included (Figure 4). Missing longitudinal data from 9 digits requiring salvage surgeries were imputed using the LOCF method. Follow-up comprised a critical 48-h postoperative period for vascular insufficiency monitoring and a definitive 7-day endpoint for survival assessment. Table 1 presents the demographics of the study population and a comparison of the CBPB and SIVA groups, showing no significant intergroup differences across all variables including age, gender, ASA classification, body mass index (BMI), type of injury, ischemic/operation time.

### 3.2. Digital Skin Temperature

**Figure 5:**
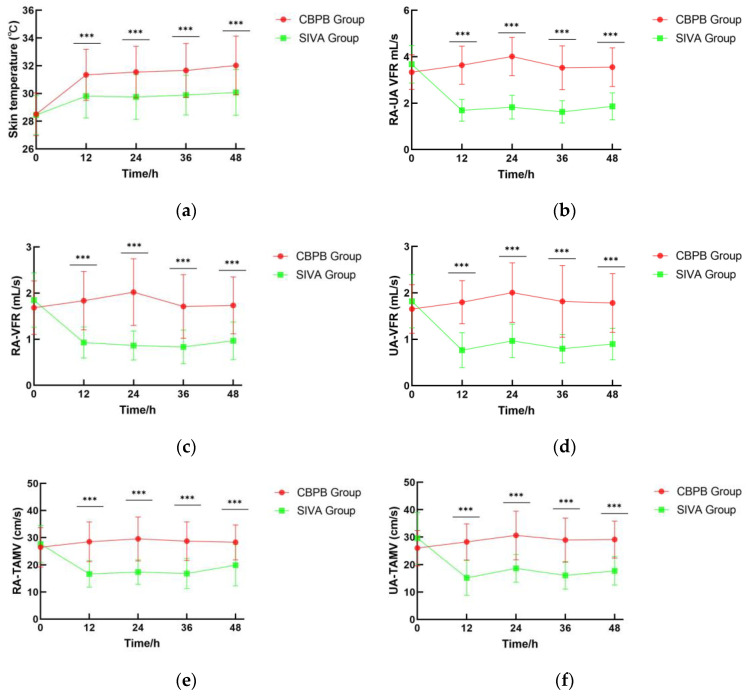

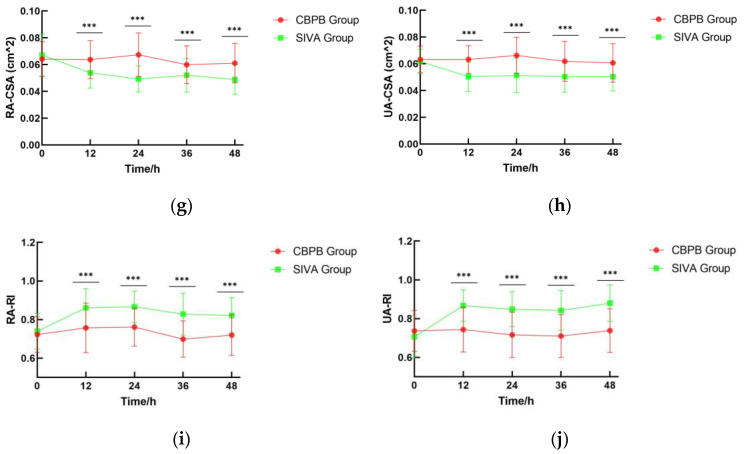
(**a**) Digit-level skin temperature and (**b**–**j**) patient-level RA-UA hemodynamics compared between CBPB (36 digits; 27 patients) and SIVA (35 digits; 28 patients). Panels b–j depict temporal changes in (**b**) RA-UA VFR, (**c**) RA-VFR, (**d**) UA-VFR, (**e**) RA-TAMV, (**f**) UA-TAMV, (**g**) RA-CSA, (**h**) UA-CSA, (**i**) RA-RI and (**j**) UA-RI. Data are presented as line graphs, with lines indicating the mean ± standard deviation. CBPB = continuous brachial plexus block; SIVA = systemic intravenous analgesia; RA-UA VFR = the combined Volumetric Flow Rate of the Radial Artery and Ulnar Artery; RA = Radial Artery; VFR = Volumetric Flow Rate; UA = Ulnar Artery; TAMV = Time-Averaged Mean Velocity; CSA = Cross-Sectional Area; RI = Resistance Index; *** *p* < 0.001.

The CBPB group maintained significantly higher skin temperatures than the SIVA group at all measured postoperative timepoints: 32 ± 1.6 °C versus 30 ± 1.1 °C at 12 h (mean difference 1.4, 95% CI 0.72 to 2.4; *p* < 0.001), 32 ± 1.5 °C versus 30 ± 1.2 °C at 24 h (mean difference 1.6, 95% CI 0.96 to 2.6; *p* < 0.001), 32 ± 1.5 °C versus 30 ± 0.79 °C at 36 h (mean difference 1.7, 95% CI 0.96 to 2.6; *p* < 0.001), and 32 ± 1.6 °C versus 31 ± 1.1 °C at 48 h (mean difference 1.8, 95% CI 1.0 to 2.8; *p* < 0.001) (Figure 5a). The Group×Time interaction was also significant (*p* < 0.001), supporting the divergent temperature trends observed in ANOVA. GEE analysis confirmed a significant group effect on skin temperature trajectory, with the CBPB group maintaining consistently higher values across all timepoints (Table 2). At the immediate postoperative phase (0 h) following recent completion of revascularization, skin temperatures in both groups remained at comparably low levels with no significant difference observed.

### 3.3. RA-UA VFR

Compared to the SIVA group, the CBPB group exhibited markedly higher RA-UA VFR at all postoperative intervals, except immediately after surgical closure (Figure 5b). Specifically, RA-UA VFR values were 3.63± 0.82 mL/s versus 1.7 ± 0.47 mL/s (mean difference 1.9, 95% CI 1.6 to 2.3; *p* < 0.001) at 12 h, 4.0 ± 0.83 mL/s versus 1.8 ± 0.51 mL/s (mean difference 2.2, 95% CI 1.8 to 2.6; *p* < 0.001) at 24 h, 3.5 ± 0.90 mL/s versus 1.6 ± 0.48 mL/s (mean difference 1.9, 95% CI 1.5 to 2.3; *p* < 0.001) at 36 h, and 3.6 ± 0.83 mL/s versus 1.9 ± 0.58 mL/s (mean difference 1.7, 95% CI 1.3 to 2.1; *p* < 0.001) at 48 h, yielding a mean intergroup difference of 1.4 mL/s. Furthermore, the CBPB group showed significant increases in CSA, TAMV, and VFR of both radial and ulnar arteries (*p* < 0.001 for all parameters), accompanied by reduced RI in these vessels (*p* < 0.001) (Figure 5c–j). No significant differences in hemodynamic parameters were observed immediately after surgery (0 h), due to residual sympatholytic effects of the initial brachial plexus blockade.

### 3.4. VAS

Within 48 h postoperatively, three SIVA patients required supplemental intravenous tramadol (300 mg, 200 mg, and 100 mg, respectively) to maintain a VAS score ≤ 3. The CBPB group demonstrated significantly superior analgesia, with a mean VAS score of 0.52 ± 0.51 versus 1.9 ± 1.0 in the SIVA group (mean difference 1.3, 95% CI: 0.89–1.8; *p* < 0.001).

### 3.5. Postoperative Incidence of Vascular Insufficiency

The postoperative incidence of vascular insufficiency and subsequent secondary revascularization were summarized in Table 3. The overall incidence of vascular insufficiency was 18% (13/71), with the CBPB group demonstrating significantly lower rates than the SIVA group (8.3% [3 of 36] versus 29% [10 of 35], risk ratio 0.29, 95% CI 0.09–0.97, *p* = 0.028; GEE-adjusted odds ratio 4.9, 95%CI 1.0–24, *p* = 0.048). Among the 13 digits with vascular insufficiency, 4 digits restored viability after emergency intervention, while secondary revascularization was required in the remaining 9 (9/71, 13%), with significantly higher rates in the SIVA group (23% [8/35]) versus the CBPB group (2.78% [1/36]; risk ratio 8.2, 95% CI 1.1–63, *p* = 0.014; GEE-adjusted odds ratio 9.1, 95%CI 1.2–71, *p* = 0.036).

### 3.6. 7-Day Survival Rate

Perfusion was restored in 5 digits following revascularization, while the remaining 4 progressed to irreversible necrosis, characterized by mottled black discoloration, absent capillary refill, and no Doppler signal. Critically, all necrotic digits had crush-avulsion injuries; conversely, digits with cut injuries achieved a 100% survival rate. Table 3 summarized the data on 7-day survival rates of replanted fingers. The overall survival rate of all replanted digits reached 94% (67 of 71), demonstrating no statistically significant difference between CBPB and SIVA groups at 7 days postoperatively (97% [35 of 36] versus 91% [32 of 35]; risk ratio 1.1, 95% CI 0.95 to 1.2, *p* = 0.329; GEE-adjusted odds ratio 0.32, 95%CI 1.2–71, *p* = 0.290).

### 3.7. Complications

Complications were analyzed separately between the CBPB group (regional analgesia via local anesthetics) and SIVA group (systemic NSAIDs/opioid intravenous analgesia). During CBPB implementation, aside from one case of catheter dislodgement, no complications—including inadequate analgesia, local anesthetic toxicity, pneumothorax, brachial plexus injury, Horner syndrome, phrenic nerve–related dyspnea, or localized infection—occurred among other patients. In the SIVA group, two patients experienced tramadol-induced nausea, which was effectively managed with intravenous ondansetron (4 mg). No respiratory depression, gastrointestinal ulceration, or renal impairment occurred. Postoperative LMWH use was uncomplicated, with no bleeding events, heparin-induced thrombocytopenia, or allergic reactions in either group.

## 4. Discussion

Post-replantation vascular insufficiency, predominantly instigated by pain-induced sympathetic vasoconstriction and microthrombosis, constitutes a major cause of necrosis in replanted digits [3,4]. CBPB serves as a dual-modality intervention providing both analgesia and sympathectomy, with documented 22–35% increases in forearm perfusion indices [9]. However, whether augmented perfusion reduces vascular insufficiency incidence and further improves survival rates remains uncertain. The current study found that CBPB significantly enhanced perfusion in replanted digits by augmenting proximal anastomotic blood flow, significantly reducing postoperative vascular insufficiency within 48 h compared to SIVA, though it failed to confer additional survival benefits.

Hand vasculature, which receives sympathetic nerve input from branches of the median and ulnar nerves, exhibits increased flow volume and flow velocity following brachial plexus anesthesia [13]. A prior prospective study using laser-Doppler flowmetry found axillary plexus anesthesia significantly boosted hand blood flow, with superficial layers increasing by 617% and deep layers by 292% [14]. The current study employs ultrasound-derived volumetric flow analysis, demonstrating that CBPB achieves a 1.4 mL/s hemodynamic advantage in hand perfusion compared to non-regional anesthesia (SIVA). More specifically, for the distal RA and UA, CBPB provided increased blood flow velocity, vascular dilation, and reduced vascular resistance. The proximal vascular anastomoses might have similarly benefited from these hemodynamic improvements, as evidenced by the enhanced perfusion observed in the replanted fingers.

The observed cutaneous temperature elevation mediated by CBPB in this study demonstrates improved perfusion in replanted digits, attributable to significantly augmented proximal anastomotic arterial flow. This hemodynamic enhancement effectively mitigates theoretical concerns regarding compromised sympathetic regulation in denervated distal vasculature, probably as proximal flow optimization overrides local adrenergic hyporesponsiveness through shear stress-mediated endothelial nitric oxide release [15]. Recently, laser Doppler flowmetry has been reported for assessing limb perfusion, offering quantifiable flow metrics. However, its limitations in tissue contact requirements, restricted spatial resolution, and motion artifacts render it suboptimal for serial replanted digit surveillance [16]. In contrast, infrared thermography enables non-contact, real-time thermal mapping [17], which underpins its selection in this study for dynamic perfusion evaluation in post-replantation care.

The pathophysiological mechanism of vascular insufficiency primarily involves vasospasm and subsequent thrombosis [4]. Beyond postoperative pain-driven sympathetic activation, digital vasospasm is exacerbated by hypothermia, inflammatory mediators (thromboxane/leukotriene), and ischemia–reperfusion injury [18]. Furthermore, the injured vessel wall exposes collagen to the flowing blood, initiating a proteolytic cascade that leads to the formation of a thrombus [19]. As early as 1978, Ketchum [20] suggested that enhancing microvascular free tissue transfer outcomes could be achieved by using agents that counteract thrombin’s effects on platelets and fibrinogen. However, the propensity of heparin/LMWH for surgical site hemorrhage [21] and dextran-induced cerebral/pulmonary edema secondary to fluid overload [22] collectively exemplify an inherent efficacy-safety paradox in conventional antithrombotic therapies. In contrast to conventional anticoagulant therapies, CBPB confers partial antithrombotic effects through sustained vasodilation and increased shear rate, which enhance endothelial nitric oxide release [15] and reduce platelet P-selectin expression [23] without prolonging coagulation time. Additionally, sympathetic blockade suppresses endothelial α2-adrenergic-mediated von Willebrand factor exocytosis [24], an antiplatelet-adhesion effect not shared by heparin or LMWH. By targeting the vasospasm-platelet adhesion axis without prolonging systemic coagulation times, CBPB complements rather than replicates traditional anticoagulation and may explain the observed reduction in early vascular insufficiency.

In the current study, all necrotic digits had crush-avulsion injuries; conversely, digits with cut injuries achieved a 100% survival rate. This observation reinforces meta-analytic evidence [25] demonstrating superior survival rates in clean-cut injuries (82%) versus crush/avulsion injuries (54%), attributable to the latter’s characteristic microvascular triad: perivascular disruption, endothelial shear stress, and capillary bed compromise. These pathomechanisms synergistically increase anastomotic technical demands and impair endogenous repair capacity. Collectively, we held the opinion that the injury mechanism serves as a critical determinant of replantation survival rates, while salvage procedures effectively rescue digit viability by addressing vascular compromise. Within this paradigm, the adjunctive application of CBPB in our study failed to confer additional survival benefits, a conclusion concordant with prior evidence [26] from a retrospective logistic regression analysis of 146 replantation cases.

VAS is a subjective measure influenced by individual perception, cultural factors, and analgesic expectations. Nevertheless, it remains a validated and widely used tool in acute pain research. To enhance measurement reliability, all participants received standardized pre-procedural instructions on using the 10-cm scale, and all assessments were conducted by trained staff under uniform conditions. Although all patients in this study achieved a postoperative VAS ≤ 3 (indicating mild pain) [27], the CBPB group exhibited superior analgesia with a significant VAS reduction of 1.34 points compared to the SIVA group. This difference exceeded the established minimal clinically important difference (MCID) threshold of 1.3 points for acute postoperative pain [28], indicating both statistical significance and clinical relevance while aligning with reduced sympathetic-driven vasospasm—a mechanism likely responsible for improved perfusion and decreased vascular insufficiency rates.

Although only 1 case (2.5%) of catheter dislodgement was identified via skin markings in this study, subtle migration may have gone undetected. A prior study showed 81.5% catheter tip displacement (mean 1 cm) in 27 interscalene blocks, highlighting the need for routine post-placement ultrasound to confirm stability [29]. No phrenic nerve–related dyspnea occurred, consistent with prior cadaveric findings [30] on ultrasound-guided costoclavicular blocks sparing this nerve. SIVA-induced nausea/vomiting disrupted replant immobilization, underscoring CBPB’s opioid-sparing benefit. Compared to heparin, LMWH has similar success rates of replantation and incidence rates of vascular insufficiency, but it is associated with lower rates of postoperative bleeding and hypocoagulability [31], providing the rationale for LMWH administration in the present study with no associated complications observed.

The present study has several limitations. First, this was a single-centre study with a relatively small sample size, and these results must be considered preliminary and need to be validated in future studies. Second, the assessment of skin color/pallor was inherently subjective. To mitigate this risk, evaluations were performed independently by two blinded microsurgical nurses with senior surgeon arbitration for disagreements; nevertheless, residual assessor bias cannot be entirely excluded for color-based parameters. Third, postoperative vascular compromise rates and survival outcomes differ significantly between children and adults [32]. While children’s stronger vascular endothelial repair contributes to higher survival rates, their smaller vessel diameter increases anastomotic difficulty and spasm susceptibility. Due to pediatric referral protocols, we excluded children, thus our findings might be inapplicable to this population. Fourth, while 7-day survival is a validated early indicator of microvascular patency, definitive success should ideally be assessed at six months or more postoperatively. Long-term survival outcomes are currently being collected and will be reported separately. Fifth, smoking increases replant failure risk through nicotine-induced vasoconstriction [33]. Despite mandatory postoperative smoking abstinence for at least one month, adverse effects from pre-existing smoking history likely persist.

## 5. Conclusions

We found that CBPB provided superior analgesic efficacy and significantly enhanced perfusion in replanted digits by augmenting proximal anastomotic blood flow, leading to a marked reduction in vascular insufficiency compared to SIVA 48-h postoperatively. We propose that CBPB’s lack of survival benefits stems from salvage procedures addressing vascular compromise, while the injury mechanism critically determines digit necrosis. Further multicenter studies with larger cohorts are warranted to validate these results and explore long-term functional outcomes.

## Figures and Tables

**Table 1:** Patient demographics and characteristics.

Variables	Total (*n* = 55, 71 Digits)	CBPB (*n* = 27, 36 Digits)	SIVA (*n* = 28, 35 Digits)	*p* Value *
Age, mean (SD), years	34 (11)	34 (11)	34 (12)	0.873
Sex, *n* (%)				
Male	43 (78)	21 (78)	22 (79)	1.000
Female	12 (22)	6 (22)	6 (21)	1.000
ASA, *n* (%)				
I	25 (45)	11 (41)	14 (50)	0.181
II	30 (55)	16 (59)	14 (50)	0.150
BMI, mean (SD), kg/m^2^	24 (1.1)	24 (1.2)	24 (0.93)	0.807
Type of injury, *n* (%)				
Cut	43/71 (61)	19/36 (53)	24/35 (69)	0.174
Crushed/Avulsion	28/71 (39)	17/36 (47)	11/35 (31)	0.174
Ischemic time † (SD), hours	6.2 (2.6)	6.5 (2.8)	5.9 (2.4)	0.408
Operation time (SD), hours	4.3 (1.5)	4.5 (1.5)	4.2 (1.4)	0.830

Abbreviations: CBPB, continuous brachial plexus block; SIVA, systemic intravenous analgesia; SD, standard deviation; ASA, American society of anesthesiologists; BMI, body mass index. Continuous variables are presented as mean (standard deviation), whilst categorical variables are given as number (percentage). * Intergroup comparison between the CBPB group and SIVA group. † Ischemic time is defined as the interval from the moment of injury to the onset of surgical tourniquet inflation.

**Table 2:** GEE-adjusted skin temperature differences between groups.

Time Point (h)	β (°C)	95% CI	Wald χ^2^	*p* Value
12	1.5	2.2 to 0.73	15.09	<0.001
24	1.7	2.5 to 1.0	21.39	<0.001
36	1.7	2.5 to 0.97	20.25	<0.001
48	1.9	2.7 to 1.1	21.69	<0.001

Abbreviations: GEE, generalized estimating equations.

**Table 3:** Postoperative outcomes.

Variables	Total (*n* = 55, 71 Digits)	CBPB (*n* = 27, 36 Digits)	SIVA (*n* = 28, 35 Digits)	*p* Value ^1^	*p* Value ^2^
Vascular insufficiency incidence	13/71(18)	3/36(8.3)	10/35(29)	0.028	0.048
Secondary revascularization	9/71(13)	1/36(2.8)	8/35(23)	0.014	0.036
Survival rate	67/71(94)	35/36(97)	32/35(91)	0.329	0.290

Abbreviations: CBPB, continuous brachial plexus block; SIVA, systemic intravenous analgesia. Categorical variables are given as number (percentage). For intergroup comparisons between the CBPB and SIVA groups, ^1^ χ^2^/Fisher’s exact tests were performed at the digit level for categorical outcomes, whereas ^2^ generalized estimating equations with an exchangeable correlation structure were employed.

## Data Availability

The original datasets generated for this study are available from the corresponding author upon reasonable request. Data sharing is subject to institutional ethical approval and participant confidentiality agreements.

## Abbreviations

The following abbreviations are used in this manuscript:

SIVA	Systemic Intravenous Analgesia
NSAIDs	Nonsteroidal Anti-Inflammatory Drugs
CBPB	Continuous Brachial Plexus Block
ASA	American Society of Anesthesiologist
BPB	Brachial Plexus Blockade
VAS	Visual Analogue Scale
LMWH	Low Molecular Weight Heparin
APTT	Activated Partial Thromboplastin Time
VFR	Volumetric Flow Rate
RA-UA VFR	the combined Volumetric Flow Rate of the Radial Artery and Ulnar Artery
RA	Radial Artery
UA	Ulnar Artery
CSA	Cross-Sectional Area
TAMV	Time-Averaged Mean Velocity
RI	Resistance Index
SD	Standard Deviation
GEE	Generalized Estimating EquationsLast
LOCF	Observation Carried Forward
BMI	Body Mass Index
MCID	Minimal Clinically Important Difference
